# Hepatic lipidomics analysis reveals the anti-obesity effects of insoluble dietary fiber from okara combined with intermittent fasting treatment in high-fat diet-fed mice

**DOI:** 10.3389/fnut.2025.1549105

**Published:** 2025-04-23

**Authors:** Chenhao Zhao, Sainan Wang, Bo Li, Zhao Zhang, Wei Yu, Hansong Yu

**Affiliations:** ^1^Department of Gastrointestinal and Colorectal Surgery, China-Japan Union Hospital of Jilin University, Changchun, China; ^2^College of Food Science and Engineering, Jilin Agricultural University, Changchun, China; ^3^Division of Soybean Processing, Soybean Research & Development Center, Chinese Agricultural Research System, Changchun, China; ^4^Shandong Sinoglory Health Food Co., Ltd., Liaocheng, China

**Keywords:** insoluble dietary fiber, okara, intermittent fasting, high-fat diet, anti-obesity, lipidomics

## Abstract

**Introduction:**

Emerging evidence has revealed that supplementation with insoluble dietary fiber (IDF) improves lipid metabolic disorders caused by a high-fat diet (HFD). Except for dietary supplementation, intermittent fasting (IF) has received widespread attention as a new dietary strategy against obesity. We hypothesized that IDF combined with IF treatment may synergistically alleviate HFD-induced lipid metabolic imbalance.

**Methods:**

This study integrated biochemical analysis with hepatic lipidomics to explore lipid biomarkers and potential mechanisms associated with the anti-obesity effects of IDF combined with IF treatment.

**Results and discussion:**

The results indicated that IDF combined with IF treatment improved metabolic parameters associated with the obesity phenotype. Lipidomics analysis revealed that IDF combined with IF treatment altered hepatic lipid metabolic patterns in HFD-fed mice. Moreover, 15 differentially regulated lipid species were identified as lipid biomarkers. Moreover, the expression of lipogenesis-, lipid oxidation-, and cholesterol metabolism-related genes was also regulated. Our results indicate that IDF combined with IF treatment ameliorates lipid metabolism disorders in HFD-fed mice by regulating hepatic lipid metabolites and related gene expression, providing evidence for its anti-obesity effects.

## 1 Introduction

Obesity has become a global epidemic as its incidence has risen at an alarming rate over the last few decades. According to the World Health Organization (WHO), more than 1.9 billion adults (39% of adults) were overweight in 2016, with more than 650 million adults (13% of adults) obese; by 2030, 51% of adults worldwide are estimated to be obese (1). Growing evidence suggests that unhealthy eating patterns and lifestyles are the leading causes of obesity (2, 3). A high-fat diet (HFD) is recognized as a high-risk driver for obesity, resulting in increased *de novo* lipogenesis, decreased fatty acid oxidation, and impaired triglyceride (TG) output. The liver plays an integral role in lipid metabolism. An imbalance of lipid metabolism leads to lipid accumulation in hepatocytes, which in turn triggers the development of complications such as nonalcoholic fatty liver disease (NAFLD) (4). To date, many strategies for treating obesity and related disorders have been developed, mainly including medication, bariatric surgery, and lifestyle intervention. However, their efficacy is frequently compromised by severe adverse reactions, inevitable side effects, and poor compliance (5, 6). Therefore, efforts have been made to generate natural bioactive compounds in foods to prevent obesity.

DF has plentiful health benefits for metabolic diseases and is classified as either soluble dietary fiber (SDF) or insoluble dietary fiber (IDF) based on its water solubility. Numerous studies have indicated that IDF plays a vital role in the prevention and management of chronic metabolic diseases, challenging the conventional view that the viscosity and solubility of SDF are the main drivers of these beneficial effects (7–9). Okara is a major by-product of soymilk and tofu production, with a huge annual yield (10, 11). Due to its rough texture and difficulty in storage, okara is usually used as animal feed or discarded, leading to significant resource waste. Therefore, the accumulation of okara is a primary challenge for the soybean processing industry. Okara is rich in dietary fiber, making it a readily available and inexpensive source of fiber. In the previous study, we reported the preparation method, monosaccharide composition, structure, and physicochemical properties of high-purity IDF from okara (HPSIDF) for the first time. Moreover, we explored the regulatory effect of HPSIDF on lipid metabolism in HFD-fed mice (12). However, there are numerous lipid classes, and many lipids have different biological activities. Thus, the specific lipids affected by HPSIDF should be accurately identified.

Aside from the anti-obesity effects of natural bioactive components in foods, intermittent fasting (IF) has received much attention as an emerging dietary strategy. IF strategies can be roughly split into two categories: time-restricted feeding (foods and beverages consumed ad libitum within specific time frames) and alternate-day fasting (alternating days of fasting with no energy-containing foods or beverages consumed on scheduled fasting days) (13–15). Alternate-day fasting is a more straightforward and easier-to-implement strategy that is more tolerable in human trials (16). This makes alternate-day fasting an attractive option for combating metabolic diseases, but the specific mechanisms behind its beneficial effects are not fully understood.

In recent years, lipidomics has emerged as an effective method for the diagnosis of lipid-related diseases (cardiovascular disease, diabetes, and obesity) by analyzing lipid composition and identifying lipid biomarkers (17–19). A significant number of lipidomic studies demonstrated that obesity is primarily associated with lipid abnormalities (e.g., TGs, phosphatidylcholines [PCs], phosphatidylserines [PSs], and phosphatidylethanolamine [PEs], etc.) in human and mouse livers (20). Another research verified that PCs, lysophosphatidylcholines (LPCs), and lysophosphatidylethanolamines (LPEs) could be biomarkers in obese-resistant mice (21). In addition, a recent study has observed that the abnormal metabolism of glycerophospholipids (GP), fatty acyls (FA), and glycerolipids (GL) is linked to obesity (22). However, there are currently no studies on the use of lipidomics to explore the efficacy of HPSIDF or to elucidate lipid biomarkers associated with HPSIDF combined with IF treatment for obesity prevention.

In this study, we preliminarily investigated whether HPSIDF combined with IF intervention has a synergistic anti-obesity effect by measuring basal metabolic parameters in high-fat-fed mice. Meanwhile, we elucidated the link between biological and metabolic phenotypes of obese mice, and the biomarkers associated with HPSIDF combined with IF intervention by capturing the changes in liposome levels. We also measured the changes in the expression levels of lipid metabolism-related genes to reveal the potential metabolic pathways associated with the synergistic anti-obesity effect of HPSIDF combined with IF, providing new insights into the potential regulatory mechanisms of HPSIDF combined with IF.

## 2 Materials and methods

### 2.1 Materials and chemicals

HPSIDF was prepared as described in our previous studies. The full details were shown in Supplementary material (12). Kits for TG, total cholesterol (TC), high-density lipoprotein cholesterol (HDL-C), and low-density lipoprotein cholesterol (LDL-C) were purchased from Nanjing Jiancheng Bioengineering Institute (Nanjing, China). Kit for free fatty acid (FFA) was purchased from Shanghai Enzyme-linked Biotechnology Co., Ltd. (Shanghai, China). HPLC-grade acetonitrile, methanol (MeOH), isopropanol, and tert-butyl methyl ether (MTBE) were purchased from Merck (Darmstadt, Germany). HPLC-grade formic acid was purchased from Sigma Aldrich (Shanghai, China). Ammonium formate was purchased from Fisher (MA, USA). Lipid standards were purchased from Sigma Aldrich or Avanti Polar Lipids (Alabaster, AL, USA). The PrimeScript™RT reagent Kit and SYBR® Premix Ex Taq™ II were purchased from Takara (Beijing, China).

### 2.2 Animal experimental design and sample collection

Fifty male C57BL/6 J mice (six-week-old, 18–20 g) were obtained from the Experimental Animal Center of Jilin Agricultural University. These mice were housed in a climate-controlled environment (temperature 23 ± 2°C, relative humidity 55 ± 5%) with a light cycle (12 h light/dark). All experimental procedures were conducted according to the Laboratory Animals of Jilin Agricultural University guidelines and approved by the Laboratory Animal Welfare and Ethics Committee of Jilin Agricultural University (No. 2019 04 10 005). After adaptive feeding for a week, the mice were randomly separated into the following five groups (*n* = 10 per group) for 18 weeks: (1) ND group, in which mice were fed a normal diet; (2) HFD group, in which mice were fed a high-fat diet; (3) IF group, in which mice were fed a high-fat diet and treatment with alternate-day fasting; (4) HPSIDF group, in which mice were fed a high-fat diet plus HPSIDF (1,000 mg/kg BW); (5) HPSIDF + IF group, in which mice were fed a high-fat diet plus HPSIDF (1,000 mg/kg BW), and treatment with alternate-day fasting. The normal diet for the ND group consisted of 10 kcal% fat, 20 kcal% protein, and 70 kcal% carbohydrates, while the high-fat diets for the HFD, IF, HPSIDF, and HPSIDF + IF groups contained 60 kcal% fat, 20 kcal% protein, and 20 kcal% carbohydrates. Both types of diets were purchased from Beijing Keao Xieli Feed Co., Ltd. (Beijing, China), and their detailed information about the specific ingredients and calorie content is shown in Supplementary Table S2. During the experiment, the mice in the HPSIDF and HPSIDF + IF groups were given HPSIDF by gavage (1,000 mg/kg BW once a day). The same volume of distilled water was given to the mice in the ND, HFD, and IF groups (1,000 mg/kg BW once a day). Food intake was recorded daily for mice in the ND, HFD, and HPSIDF groups and on non-fasting days for mice in the IF and HPSIDF + IF groups. Body weight was recorded weekly, and the final body length was measured to calculate Lee’s index as [body weight (g)^1/3^ × 1,000/body length (cm)] (23–25). At the end of the experimental period, all mice were fasted for 12 h and then anesthetized. Blood samples were collected from the eyeball and then centrifuged at 3,000 g for 15 min at 4°C to obtain serum. The liver and adipose tissues were obtained, weighed, and immediately stored at −80°C for further analysis.

### 2.3 Biochemical analysis

The levels of serum TG, TC, HDL-C, LDL-C, and hepatic TG and TC were analyzed by an enzymatic method using commercial kits. The ELISA kit determined the levels of FFA according to the manufacturer’s instructions.

### 2.4 Histological analysis

The procedures of hematoxylin and eosin (H&E) staining were reported in our previous studies (12). Briefly, the collected liver tissues were fixed in 4% paraformaldehyde, embedded in paraffin to prepare tissue sections (5 μm thickness), and stained with hematoxylin and eosin (H&E). Moreover, Oil red O staining was used to analyze lipid droplet accumulation in frozen liver sections. Images were acquired with an optical microscope (Olympus, Tokyo, Japan) to assess pathological differences among the liver tissues from each group.

### 2.5 Hepatic lipidomics analysis

Liver tissue samples (20 mg) were harvested from the ND, HFD, IF, HPSIDF, and HPSIDF + IF groups (*n* = 4) and transferred to EP tubes. The samples were homogenized (30 HZ) for 20 s with a steel ball and centrifuged (3,000 rpm, 4°C) for 30 s. Then, 1 mL of the extraction solvent (MTBE/MeOH = 3:1, v/v) containing an internal standard mixture was added to the samples. After whirling the mixture for 15 min, 200 μL of water was added. After homogenization, the mixture was vortexed for 1 min and then centrifuged at 12,000 rpm for 10 min. Next, 200 μL of the upper organic layer was collected from each sample and evaporated using a vacuum concentrator at 37°C. Finally, the lipid extracts were redissolved in 200 μL of mobile phase B for LC–MS/MS analysis.

Hepatic lipidomics analysis was performed using an LC-ESI-MS/MS system (UPLC, ExionLC AD;^1^ MS, QTRAP® System, see text footnote 1). The sample extracts (2 μL) were separated on a Thermo Accucore™ C30 column (2.6 μm, 2.1 mm × 100 mm i.d., Sunnyvale, CA, USA) with a flow rate of 0.35 mL/min and a column temperature of 45°C. The mobile phases consisted of a mixture of acetonitrile/water (60:40, v/v) (A) and a mixture of acetonitrile/isopropanol (10:90, v/v) (B), both containing 0.1% acetic acid and 10 mmol/L ammonium formate. The solvent gradient program was as follows: 0–15.5 min, 20–95% B; 15.5–17.3 min, 95% B; 17.3–17.5 min, 95–20% B; and 17.5–20 min, 20% B. The effluent was alternatively connected to an ESI-triple quadrupole-linear ion trap (QTRAP)-MS. Shanghai Personalbio Technology Co., Ltd., carried out the qualitative and quantitative analyses of lipid profiling.

### 2.6 Quantitative reverse transcription PCR (qRT-PCR)

Total RNA was extracted from the frozen liver tissues using Trizol reagent and reverse-transcribed to cDNA by a PrimeScript™RT reagent kit with a gDNA Eraser. qRT-PCR assays were performed on Biometra TProfessional PCR (Jena, Germany) using SYBR® Premix Ex Taq™ II. The thermal cycle conditions were initiated at 95°C for 30 s and 40 cycles of amplification (95°C for 10 s, 56°C for 30 s, and 72°C for 30 s). The expression of target genes was reported relative to the internal control (β-actin) and was calculated using the 2^–ΔΔCT^ method. The primer sequences of genes analyzed by qRT-PCR are listed in Supplementary Table S3.

### 2.7 Statistical analysis

All data were presented as mean ± standard deviation (SD) and analyzed using SPSS 19.0 (SPSS, Chicago, IL, USA). The statistical significance between groups was determined by one-way analysis of variance (ANOVA) with Duncan’s multiple range tests. The differences were considered significant at *p* < 0.05. The lipid data were analyzed using the MetaboAnalystR 1.0.1 online software.^2^ The significantly differential lipid species among experimental groups were determined by variable importance in the projection (VIP ≥ 1) from the orthogonal partial least-squares discriminant analysis (OPLS-DA) model and fold change (FC ≥ 2 or ≤0.5) (26, 27).

## 3 Results

### 3.1 Effects of HPSIDF combined with IF treatment on body weight gain, food efficiency ratio, and fat accumulation in HFD-fed mice

After 18 weeks of feeding, the body weight gain of mice in the HFD group was remarkably higher than that in the ND group (*p* < 0.05) (Table 1). However, the IF, HPSIDF, and HPSIDF + IF groups significantly inhibited high-fat diet-induced weight gain compared with the HFD group, especially the HPSIDF + IF group (*p* < 0.05). Interestingly, the food intake of the IF group did not show a statistically significant difference compared to that of the ND and HFD groups. Based on this observation, we hypothesized that alternate-day fasting would not affect the mice’s appetite on non-fasting days. Furthermore, the decrease in food intake in the HPSIDF and HPSIDF + IF groups may be due to the satiating effect of HPSIDF. Importantly, as shown in Supplementary Table S2, the energy density of the high-fat diet was significantly higher than that of the normal diet. Thus, the energy intake of mice in the IF group on non-fasting days was equal to that of the HFD group and remarkably higher than that of the ND group (*p* < 0.05). Moreover, energy intake was lower in the HPSIDF and HPSIDF + IF groups than in the HFD group (*p* < 0.05). Similar results were also found in the food efficiency ratio. Lee’s index is a valuable tool for determining the degree of obesity (28–30). The HPSIDF + IF group dramatically reduced Lee’s index of HFD-fed mice compared to the IF and HPSIDF groups.

**Table 1:** Effects of HPSIDF combined with IF treatment on body weight, energy intake, and food efficiency ratio in mice fed with HFD.^a^

Items	ND	HFD	IF	HPSIDF	HPSIDF + IF
Initial body weight (g)	21.02 ± 0.68	21.60 ± 1.09	21.70 ± 0.94	21.65 ± 0.73	21.67 ± 0.53
Final body weight (g)	28.43 ± 0.88^c^	35.13 ± 0.82^a^	31.05 ± 0.85^b^	29.65 ± 1.31^c^	27.52 ± 0.97^c^
Weight gain (g)	7.41 ± 0.88^c^	13.53 ± 0.82^a^	9.35 ± 0.85^b^	8.00 ± 1.31^c^	5.85 ± 0.97^d^
Food intake (g/day per mouse)	2.91 ± 0.16^a^	2.70 ± 0.16^b^	2.81 ± 0.12^ab^	2.32 ± 0.14^c^	2.36 ± 0.15^c^
Energy intake (kcal/day per mouse)	11.18 ± 0.60^b^	14.75 ± 0.64^a^	14.12 ± 0.83^a^	12.39 ± 0.80^b^	12.00 ± 0.68^b^
Food efficiency ratio (%)^b^	0.53 ± 0.03^b^	0.73 ± 0.03^a^	0.53 ± 0.03^b^	0.51 ± 0.03^b^	0.39 ± 0.02^c^
Lee’s index	311.48 ± 7.56^b^	331.44 ± 7.62^a^	323.98 ± 6.22^a^	314.36 ± 6.66^b^	308.77 ± 5.29^b^

a HPSIDF, high-purity insoluble dietary fiber from okara; IF, intermittent fasting; HFD, high-fat diet.

b Food efficiency ratio (FER) = body weight gain (g)/energy intake (kcal/day per mouse).

Different letters in the same line (a, b, c, and d) are significantly different (*p* < 0.05). Results are expressed as mean ± SD (*n* = 10).

Furthermore, we observed that the HFD group exhibited a significantly higher relative weight of liver, epididymal, subcutaneous, and groin adipose tissues in contrast with the ND group (*p* < 0.05) (Table 2). The liver weight of mice was significantly lower in the IF, HPSIDF, and HPSIDF + IF groups compared to the HFD group (*p* < 0.05), and there was no significant difference between the groups (*p* > 0.05). Moreover, IF, HPSIDF and HPSIDF combined with IF treatment significantly reduced the relative weight gain of the epididymal, subcutaneous, and groin adipose tissues induced by HFD (*p* < 0.05). The relative weight of epididymis, subcutaneous and groin adipose tissue was remarkably reduced in mice in the HPSIDF + IF group compared to the IF group (*p* < 0.05). Meanwhile, the HPSIDF combined with IF treatment dramatically decreased the weight of groin adipose tissue compared to the HPSIDF group (*p* < 0.05). Interestingly, when compared with the HFD group, the HPSIDF + IF group markedly increased the relative weight of scapular adipose tissue (*p* < 0.05). These results demonstrated that HPSIDF combined with IF treatment could effectively reduce body weight gain and fat accumulation in HFD-fed mice.

**Table 2:** Effects of HPSIDF combined with IF treatment on liver index and relative adipose tissue weight in mice fed with HFD.^a^

Items^b^	ND	HFD	IF	HPSIDF	HPSIDF + IF
Liver index (%)	3.42 ± 0.11^b^	3.68 ± 0.09^a^	3.51 ± 0.11^b^	3.45 ± 0.12^b^	3.41 ± 0.06^b^
Epididymal fat (%)	0.74 ± 0.09^d^	1.87 ± 0.30^a^	1.52 ± 0.31^b^	1.30 ± 0.26^bc^	1.09 ± 0.14^c^
Subcutaneous fat (%)	2.10 ± 0.18^c^	4.99 ± 1.07^a^	3.49 ± 0.28^b^	2.79 ± 0.31^bc^	2.42 ± 0.23^c^
Groin fat (%)	0.22 ± 0.05^e^	0.75 ± 0.08^a^	0.55 ± 0.04^b^	0.47 ± 0.02^c^	0.37 ± 0.03^d^
Scapular fat (%)	0.21 ± 0.01^d^	0.18 ± 0.02^c^	0.22 ± 0.02^c^	0.27 ± 0.02^b^	0.32 ± 0.05^a^

a HPSIDF, high-purity insoluble dietary fiber from okara; IF, intermittent fasting; HFD, high-fat diet.

b Liver index (%) = liver weight (g)/body weight (g).

The relative adipose tissue weight was calculated as a percentage of body weight [e.g., relative epididymal fat weight (%) = epididymal fat weight (g)/final body weight (g)]. Different letters in the same line (a, b, c, d, and e) are significantly different (*p* < 0.05). Results are expressed as mean ± SD (*n* = 10).

### 3.2 Effects of HPSIDF combined with IF treatment on biochemical parameters in HFD-fed mice

As shown in Table 3, the levels of serum TG, TC, LDL-C, and FFA were significantly increased in the HFD group when compared with the ND group. In contrast, HPSIDF combined with IF treatment inhibited the increase of these indicators (*p* < 0.05). Besides, the serum HDL-C levels of mice in the HFD group were reduced compared with the ND group (*p* < 0.05). As expected, HPSIDF combined with IF treatment markedly enhanced serum HDL-C levels (*p* < 0.05). Additionally, consistent with the results of lipid levels in serum, HPSIDF combined with IF treatment dramatically reduced TG and TC levels in the liver of HFD-fed mice (*p* < 0.05).

**Table 3:** Effects of HPSIDF combined with IF treatment on the lipid levels of mice fed with HFD.^a^

Parameters	ND	HFD	IF	HPSIDF	HPSIDF + IF
Serum TC (mmol/L)	5.62 ± 0.20^b^	7.40 ± 0.34^a^	5.98 ± 0.48^b^	5.40 ± 0.47^b^	5.34 ± 0.51^b^
Serum TG (mmol/L)	0.93 ± 0.05^b^	1.17 ± 0.16^a^	0.83 ± 0.11^bc^	0.75 ± 0.11^cd^	0.61 ± 0.08^d^
Serum LDL-C (mmol/L)	0.41 ± 0.08^c^	1.51 ± 0.42^a^	0.94 ± 0.19^b^	0.57 ± 0.11^c^	0.45 ± 0.08^c^
Serum HDL-C (mmol/L)	4.58 ± 0.57^a^	2.86 ± 0.53^b^	4.15 ± 0.72^ab^	4.36 ± 0.55^a^	4.38 ± 0.75^a^
Serum FFA (μmol/L)	79.61 ± 7.11^c^	124.92 ± 9.71^a^	115.81 ± 10.88^a^	95.41 ± 4.34^b^	83.51 ± 6.71^bc^
Liver TC (mmol/g prot)	1.19 ± 0.08^c^	1.85 ± 0.24^a^	1.55 ± 0.12^b^	1.34 ± 0.06^bc^	1.26 ± 0.11^c^
Liver TG (mmol/g prot)	1.58 ± 0.29^c^	2.78 ± 0.30^a^	2.37 ± 0.11^b^	1.80 ± 0.12^c^	1.50 ± 0.15^c^

a HPSIDF, high-purity insoluble dietary fiber from okara; IF, intermittent fasting; HFD, high-fat diet.

Different letters in the same line (a, b, c, and d) are significantly different (*p* < 0.05). Results are expressed as mean ± SD (*n* = 10).

### 3.3 Effects of HPSIDF combined with IF treatment on hepatic histopathology in HFD-fed mice

**Figure 1:**
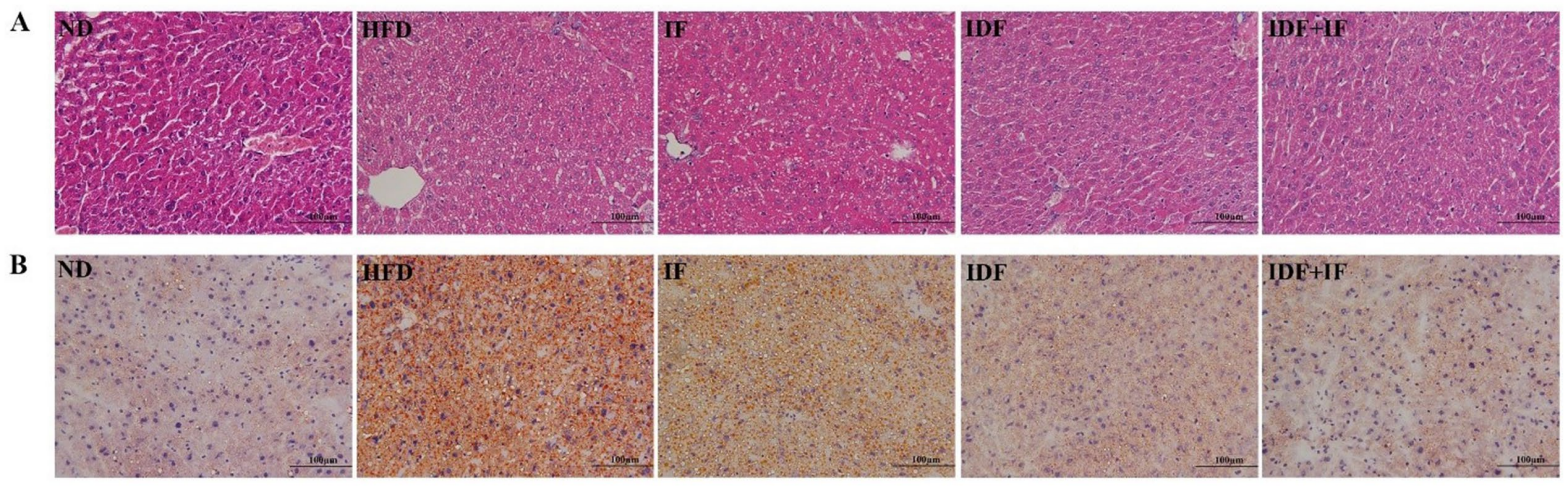
Effects of HPSIDF combined with IF treatment on hepatic histopathology in mice fed with HFD. The histological changes of liver sections were measured by H&E staining **(A)** and Oil Red O staining **(B)**. IDF represents the HPSIDF group and IDF + IF represents the HPSIDF + IF group in the figure. ND, normal diet; HFD, high-fat diet; IF, intermittent fasting; HPSIDF, high-purity insoluble dietary fiber from okara.

A marked increase in lipid droplet accumulation was observed in the liver tissue of HFD-fed mice, as determined by H&E and Oil Red O staining (Figures 1A,B). Nevertheless, the degree of lipid deposition and vacuolation in the liver sections of the HPSIDF + IF group was markedly reduced compared with the HFD group. Our findings indicated that HPSIDF combined with IF treatment could effectively reduce lipid droplet accumulation in the liver of mice induced by HFD.

### 3.4 Lipidomics analysis of hepatic lipid metabolism following HPSIDF combined with IF treatment

**Figure 2:**
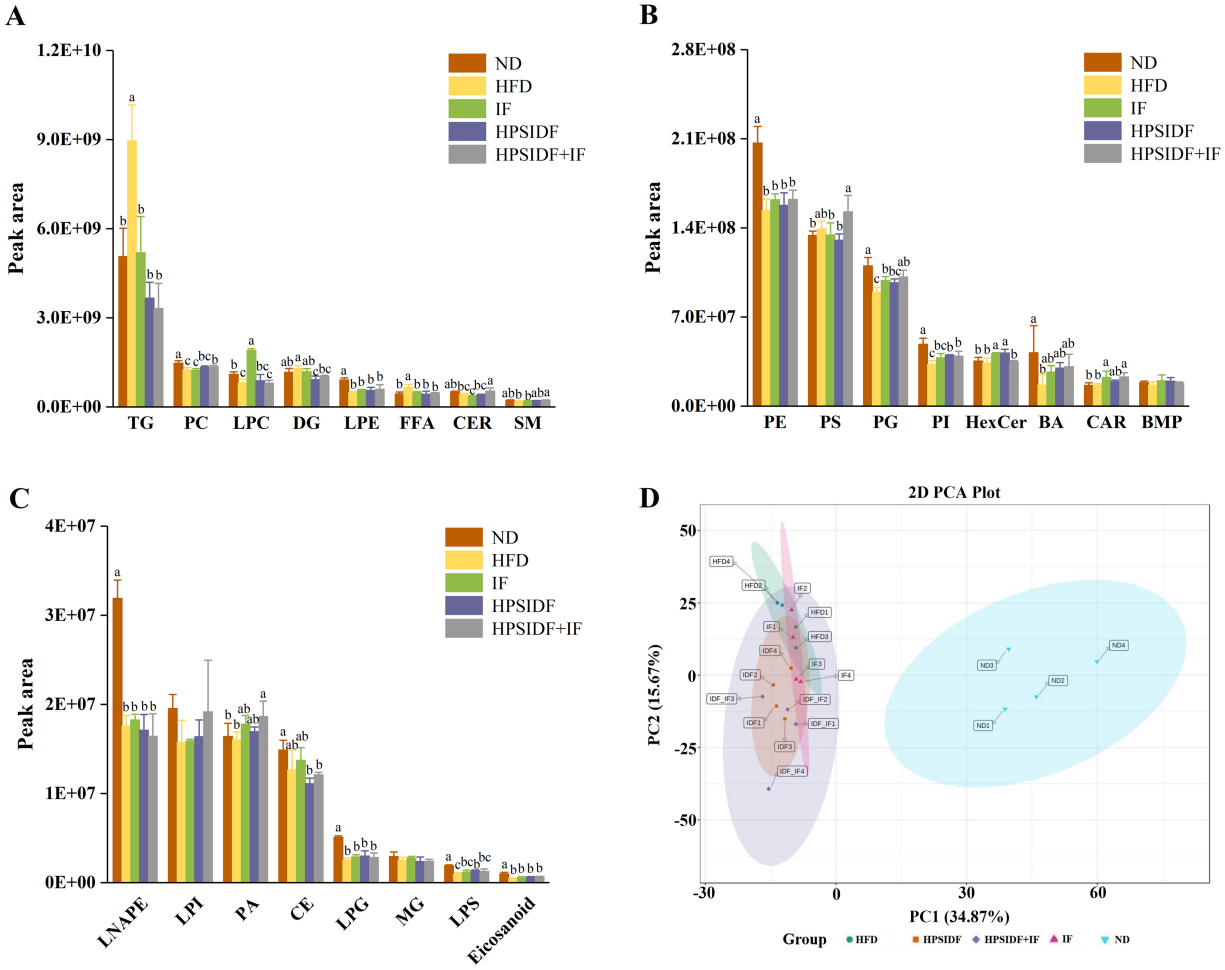
Lipidomics analysis of liver samples. The intensity of different lipid compositions in the liver **(A–C)**. Principal component analysis (PCA) score plots of hepatic lipid profiling among all groups **(D)**. IDF represents the HPSIDF group and IDF_IF represents the HPSIDF + IF group in the PCA score plot. Data are presented as mean ± SD (*n* = 4). Different letters indicate statistically significant differences (*p* < 0.05). HPSIDF, high-purity insoluble dietary fiber from okara; IF, intermittent fasting; TG, triacylglycerol; PC, phosphatidylcholine; LPC, lysophosphatidylcholine; DG, diacylglycerol; LPE, lysophosphatidylethanolamine; FFA, free fatty acid; CER, ceramide; SM, sphingomyelin; PE, phosphatidylethanolamine; PS, phosphatidylserine; PG, phosphatidylglycerol; PI, phosphatidylinositol; HexCer, hexosylceramide; BA, bile acid; CAR, carnitine; BMP, bismonoacylglycerophosphate; LNAPE, N-acyl-lysophosphatidylethanolamine; LPI, lysophosphatidylinositol; PA, phosphatidic acid; CE, cholesteryl ester; LPG, lysophosphatidylglycerol; MG, monoacylglycerol; LPS, lysophosphatidylserine.

Hepatic lipidomics profiles from mice in four experimental groups were obtained and analyzed to characterize the hepatic lipidome of HFD-fed mice further and identify potential lipid biomarkers associated with HPSIDF combined with IF treatment. A total of 1,622 lipid metabolites were identified in the liver tissues from the ND, HFD, IF, HPSIDF, and HPSIDF + IF groups, including 316 TGs, 115 diacylglycerols (DGs), 38 FFAs, 52 carnitines (CARs), 47 ceramides (CERs), 36 sphingomyelins (SMs), 151 PCs, 94 PEs, 105 phosphatidylglycerols (PGs), 74 phosphatidylinositols (PIs), 110 PSs, 52 phosphatidic acids (PAs), 45 LPCs, and others. A profound perturbation of lipid metabolism was observed in the HFD group, which exhibited higher levels of TGs, DGs, and FFAs and lower levels of PCs, PGs, PIs, CERs, SMs, BAs, and lysophosphatidylserines (LPSs) compared to the ND group. To emphasize, the intervention of HPSIDF combined with IF ameliorated the disturbance of lipid metabolism caused by HFD, which was more effective than the IF and HPSIDF groups. (Figures 2A–C). We used principal component analysis (PCA) to explore and compare variation among groups. The separation between the ND and HFD groups could be observed from the PCA score plot (Figure 2D), while the samples in the HFD and IF, HPSIDF, and HPSIDF + IF groups were dispersed on the left region of the plot and partially overlapped. Furthermore, the heatmap indicated that the lipid metabolism patterns in the HFD group were different from those in the ND group, but the metabolic patterns of the IF, HPSIDF, and HPSIDF + IF groups gradually tended to the ND group, especially the HPSIDF + IF group (Supplementary file 1).

### 3.5 HPSIDF combined with IF treatment differentially regulates hepatic lipid species

**Figure 3:**
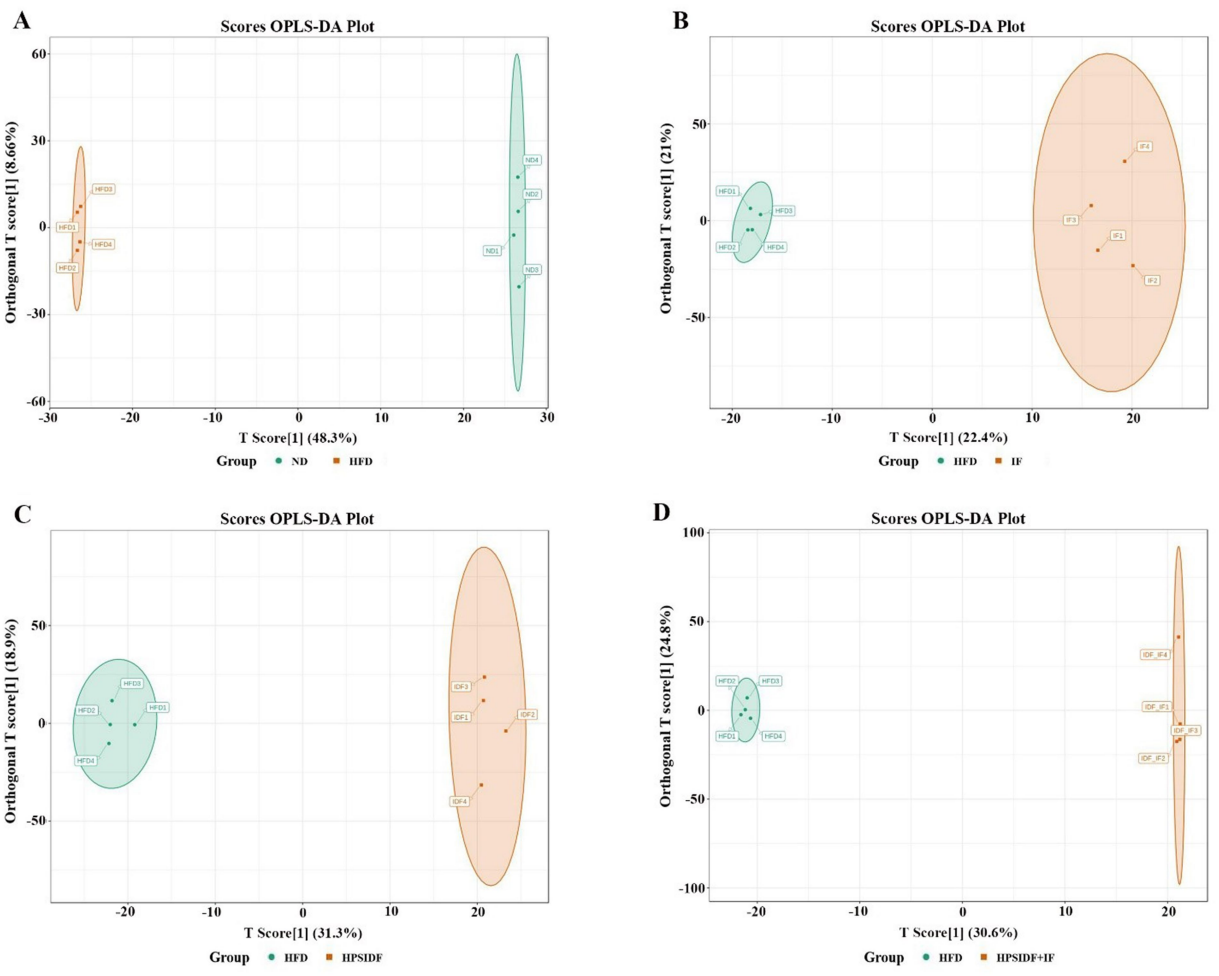
OPLS-DA scores plot analysis of HFD versus ND **(A)**, IF versus HFD **(B)**, HPSIDF versus HFD **(C)**, and HPSIDF + IF versus HFD **(D)**. IDF represents the HPSIDF group and IDF_IF represents the HPSIDF + IF group in the OPLS-DA score plots. ND, normal diet; HFD, high-fat diet; IF, intermittent fasting; HPSIDF, high-purity insoluble dietary fiber from okara.

**Figure 4:**
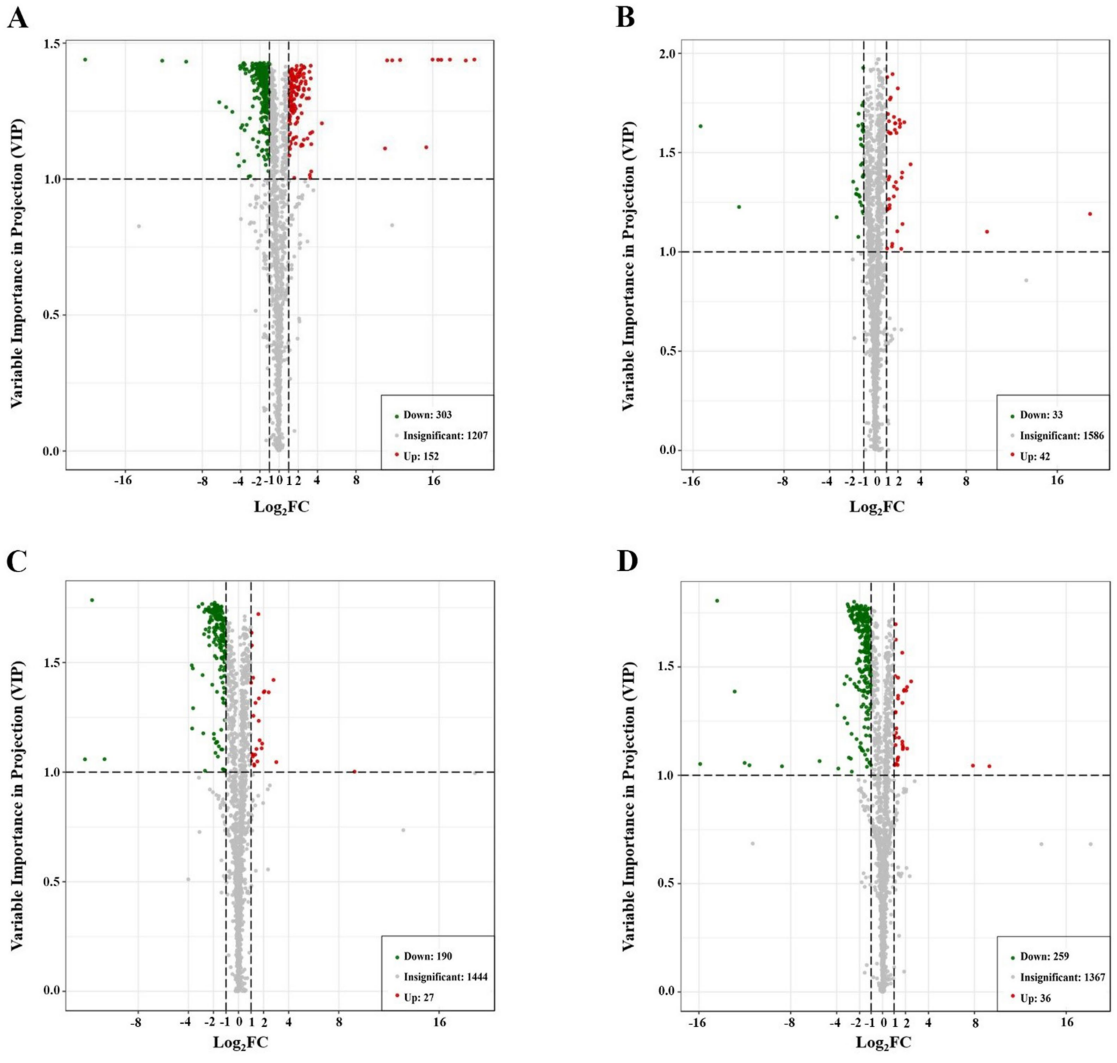
Analysis of significantly differential lipid species in HFD versus ND **(A)**, IF versus HFD **(B)**, HPSIDF versus HFD **(C)**, and HPSIDF + IF versus HFD **(D)**. In the volcano plot, a number of significantly differential lipid species were screened out based on the criteria of FC ≥ 2 or ≤0.5 and VIP ≥ 1. Significantly differential lipid species were shown as a red (up) or green (down) dot, whereas a gray dot represented no significant difference in lipid species. Data are presented as mean ± SD (*n* = 4). ND, normal diet; HFD, high-fat diet; IF, intermittent fasting; HPSIDF, high-purity insoluble dietary fiber from okara.

To achieve better separation between all experimental groups, we obtained four separate multivariate OPLS-DA models (HFD group vs. ND group, IF group vs. HFD group, HPSIDF group vs. HFD group, and HPSIDF + IF group vs. HFD group) (Figures 3A–D). The validation parameters were as follows: fitness (R^2^X = 0.569 and R^2^Y = 1) and predictability (Q^2^ = 0.967) in HFD group versus ND group model; fitness (R^2^X = 0.434 and R^2^Y = 0.995) and predictability (Q^2^ = 0.636) in IF group versus HFD group model; fitness (R^2^X = 0.502 and R^2^Y = 0.997) and predictability (Q^2^ = 0.888) in HPSIDF group versus HFD group model; fitness (R^2^X = 0.661 and R^2^Y = 1) and predictability (Q^2^ = 0.884) in HPSIDF + IF group versus HFD group model. The results showed that the goodness of fit of the OPLS-DA models was adequate for evaluating variance in lipid metabolites. Clear separations were observed in both the HFD group versus ND group model, the IF group versus HFD group model, the HPSIDF group versus HFD group model, and the HPSIDF + IF group versus HFD group model, indicating that disordered lipid metabolism was induced by HFD and intervened by the IF, HPSIDF, and HPSIDF + IF groups, especially the HPSIDF + IF group. Volcano plot analysis was used to screen out lipid biomarker candidates for HPSIDF combined with IF treatment. Using the criteria of FC ≥ 2 or ≤0.5 and VIP ≥ 1, 455 differentially regulated lipid species were identified between the ND and HFD groups (Figure 4A). Moreover, 75 differentially regulated lipid species were significantly changed between the HFD and IF groups. In comparison, 217 differentially regulated lipid species were changed considerably between the HFD and HPSIDF groups (Figures 4B,C). Notably, 295 differentially regulated lipid species significantly changed between the HFD and HPSIDF + IF groups, most of which were remarkably downregulated (Figure 4D). Therefore, HPSIDF combined with IF treatment had a greater effect on the pattern of hepatic lipid metabolism in HFD-fed mice compared to IF or HPSIDF treatment.

**Figure 5:**
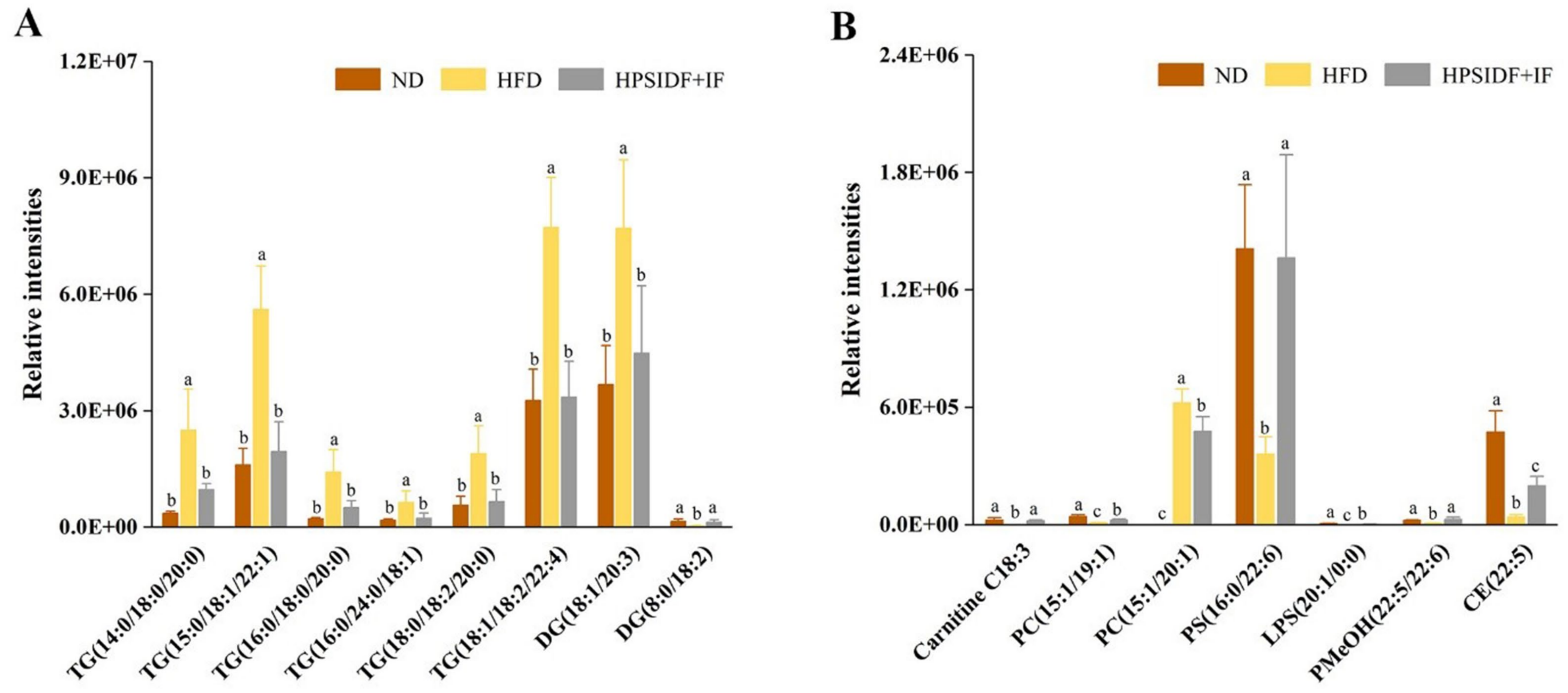
Potential lipid biomarkers in response to the effects of HPSIDF combined with IF treatment were screened out by using the set criteria (FC ≥ 2 or ≤0.5 and VIP ≥ 1) **(A,B)**. ND, normal diet; HFD, high-fat diet; IF, intermittent fasting; HPSIDF, high-purity insoluble dietary fiber from okara.

Based on these results, we further screened significant differential lipid species associated with HPSIDF combined with IF treatment as potential lipid biomarkers based on set criteria (FC ≥ 2 or ≤0.5, VIP ≥ 1). Fifteen differentially regulated lipid species, including six TGs, two DGs, one CAR, two PCs, one PS, one LPS, one PMeOH, and one CE, were identified from the ND, HFD, and HPSIDF + IF groups. As shown in Figures 5A,B, the levels of TG (14:0/18:0/20:0), TG (15:0/18:1/22:1), TG (16:0/18:0/20:0), TG (16:0/24:0/18:1), TG (18:0/18:2/20:0), TG (18:1/18:2/22:4), DG (18:1/20:3), and PC (15:1/20:1) in the HFD group were significantly increased in comparison with the ND group (*p* < 0.05), whereas the levels of these lipid species were reduced after treatment of HPSIDF combined with IF (*p* < 0.05). Besides, the levels of DG (8:0/18:2), Carnitine C18:3, PC (15:1/19:1), PS (16:0/22:6), LPS (20:1/0:0), PMeOH (22:5/22:6), and CE (22:5) were decreased in the HFD group when compared with the ND group. However, levels of these lipids were increased in the HPSIDF + IF group. Thus, 15 lipid species, including six TGs, two DGs, one CAR, two PCs, one PS, one LPS, one PMeOH, and one CE, might be considered lipid biomarkers of HPSIDF combined with IF intervention.

### 3.6 Pathway analysis

**Figure 6:**
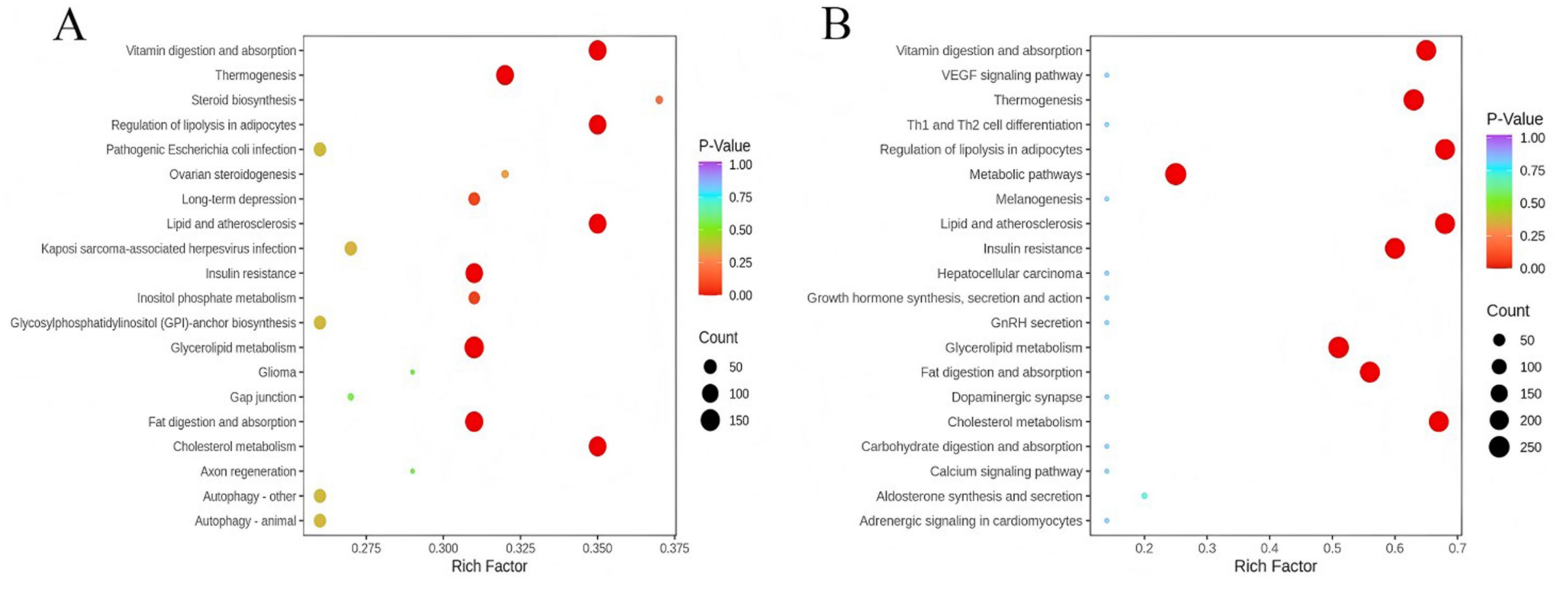
Lipid metabolic pathway analysis based on significantly differential lipid species in HFD versus ND **(A)** and HPSIDF + IF versus HFD **(B)**. The degree of enrichment was analyzed by a rich factor, *p*-value, and the number of lipid metabolites enriched in each pathway. The size of the bubble means the amount of significantly differential lipid species that are enriched in this pathway, and the point with a different gradation of color represents the scope of the *p*-value. The higher value of the rich factor stands for the higher degree of enrichment, and the lower *p*-value represents the more significant degree of enrichment. HPSIDF, high-purity insoluble dietary fiber from okara; IF, intermittent fasting; HFD, high-fat diet.

We performed pathway enrichment analysis to identify alterations in key pathways associated with HPSIDF combined with IF treatment. As depicted in Figure 6, the larger the rich factor value, the greater the enrichment degree of the metabolic pathways. The mice fed with HFD markedly affected the pathways of GL metabolism, cholesterol metabolism, fat digestion and absorption, insulin resistance, thermogenesis, and regulation of lipolysis in adipocytes as contrasted with the ND group. In contrast, HPSIDF combined with IF treatment ameliorated many of these changes.

### 3.7 Association between lipid biomarkers and obesity phenotypes

**Figure 7:**
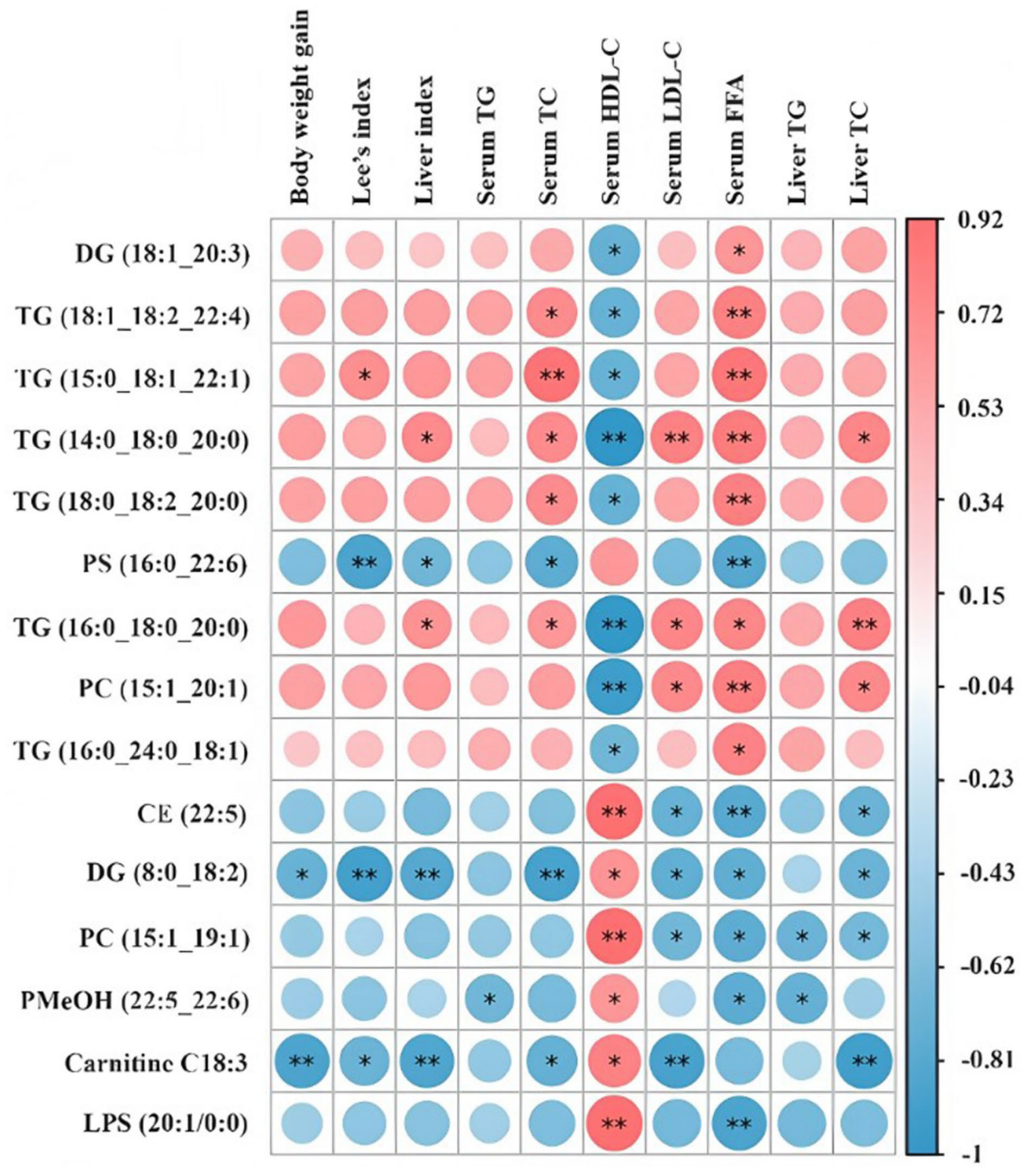
Spearman’s correlations between lipid markers and important metabolic parameters related to obesity in the ND, HFD, and HPSIDF + IF groups. HPSIDF, high-purity insoluble dietary fiber from okara; IF, intermittent fasting; HFD, high-fat diet; ND, normal diet.

Spearman’s correlation heatmap was applied to reveal the correlations between lipid biomarkers and critical metabolic parameters associated with obesity in the context of HPSIDF combined with IF treatment (Figure 7). TG (18:1/18:2/22:4), TG (15:0/18:1/22:1), TG (14:0/18:0/20:0), and TG (18:0/18:2/20:0) exhibited a positive correlation with serum TC and FFA and a negative correlation with serum HDL-C. Moreover, TG (16:0/18:0/20:0) and PC (15:1/20:1) were positively correlated with serum LDL-C and FFA, liver TC, and negatively correlated with serum HDL-C. In contrast, CE (22:5) and PC (15:1/19:1) were positively correlated with serum HDL-C and negatively correlated with serum LDL-C and FFA, and liver TC. Furthermore, DG (8:0/18:2) and Carnitine C18:3 displayed a positive correlation with serum HDL-C and a negative correlation with body weight gain, Lee’s index, liver index, serum TC and LDL-C, liver TC. Hence, there is a correlation between the above lipid biomarkers associated with HPSIDF combined with IF treatment and obesity phenotype. In future studies, we can attempt to indirectly prevent obesity by improving the levels of the above lipid biomarkers through HPSIDF combined with IF treatment.

### 3.8 Effect of HPSIDF combined with IF treatment on expression levels of genes related to hepatic lipid metabolism

**Figure 8:**
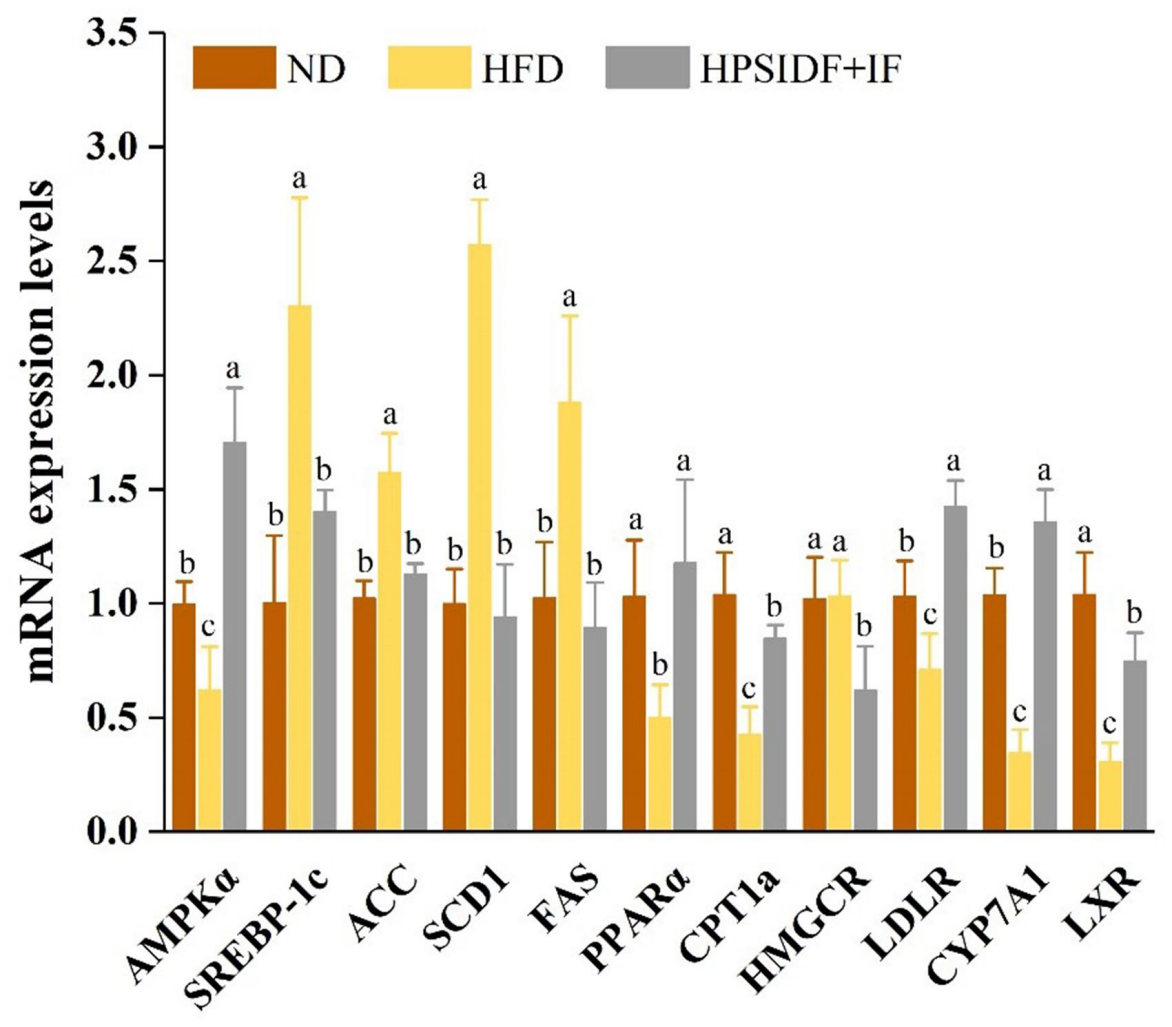
Effects of HPSIDF combined with IF treatment on mRNA expression levels of hepatic lipid metabolism genes in the ND, HFD, and HPSIDF + IF groups. Groups with different letters represent statistically significant differences (*p* < 0.05). HPSIDF, high-purity insoluble dietary fiber from okara; IF, intermittent fasting; HFD, high-fat diet; ND, normal diet.

To explore the underlying mechanism by which HPSIDF combined with IF treatment improves abnormal lipid metabolism, we analyzed the expression levels of genes involved in lipid metabolism (Figure 8). Compared with the ND group, the expression levels of adipogenic genes such as sterol regulatory element-binding protein-1c (SREBP-1c), acetyl-CoA carboxylase (ACC), fatty acid synthase (FAS), and stearoyl-CoA desaturase-1 (SCD1) were significantly up-regulated in the HFD group (*p* < 0.05). As expected, SREBP-1c, ACC, FAS, and SCD1 expression levels in the HPSIDF + IF group were remarkably down-regulated compared with the HFD group (*p* < 0.05). In addition, the expression levels of lipolytic genes such as AMP-activated protein kinase-α (AMPKα), peroxisome proliferators-activated receptor-α (PPARα), and carnitine palmitoyltransferase-1a (CPT1a) were significantly enhanced in the HPSIDF + IF group in comparison with the HFD group (*p* < 0.05). Furthermore, contrasted with the HFD group, HPSIDF combined with IF treatment significantly up-regulated the expression levels of low-density lipoprotein receptor (LDLR), cholesterol 7α-hydroxylase (CYP7A1), and liver X receptor (LXR) involved in cholesterol metabolism but down-regulated the expression levels of HMG-CoA reductase (HMGCR).

## 4 Discussion

Recently, several studies have suggested that IDF has a vital role in preventing obesity. In previous experiments, we investigated the regulatory effect of HPSIDF on HFD-induced lipid metabolic disruptions. Although HPSIDF has been to reduce blood lipid levels and alleviate hepatic steatosis, the mechanism behind the regulation of lipid homeostasis remains unknown (12). Furthermore, IF has gained popularity as an effective dietary intervention to address the difficulties of traditional calorie-restricted weight loss regimens. Based on previous data, we speculate that HPSIDF combined with IF treatment may have a more significant anti-obesity effect. In terms of metabolic parameters, we found that HPSIDF combined with IF treatment not only remarkably reduced body weight gain, fat accumulation, serum TG, TC, LDL-C, and FFA levels with enhanced serum HDL-C levels but also ameliorated hepatic steatosis by decreasing the relative liver weight, hepatic TG and TC levels, which was superior to HPSIDF and IF intervention alone. Thus, we used lipidomics and qRT-PCR analyses to explore further changes in the lipid metabolite patterns associated with HPSIDF combined with IF treatment and changes in the expression levels of genes involved in lipid metabolism.

Inefficient lipid metabolism is now well recognized as a hallmark of dysregulated lipid metabolism, which leads to the development of metabolic diseases such as obesity (26, 31). The liver, the primary organ closely related to lipid metabolism, maintains lipid homeostasis by regulating lipid synthesis and decomposition. Lipidomics has recently emerged as a powerful tool to diagnose lipid metabolism-related diseases by analyzing lipid composition and identifying lipid biomarkers (32). Therefore, applying lipidomic analysis in this study may provide new insights into investigating hepatic lipids in obesity, as well as the molecular mechanisms involved in the anti-obesity effect of HPSIDF combined with IF treatment. Analysis of total lipid contents in the liver demonstrated that TGs, DGs, and FFAs in the HFD group were remarkably enhanced compared with the ND group. Feng et al. also observed this in the livers of NAFLD patients (33). In addition to TGs and DGs accumulation, several phospholipid species, including PCs, PGs, PIs, and LPSs, were also altered in the liver of HFD-fed mice. Our results were consistent with the report of Eisinger et al. (20) that the contents of PCs, PIs, and LPSs in the fatty liver showed a downward trend. Furthermore, PG is an important precursor for the synthesis of cardiolipin, and its deficiency leads to significantly impaired mitochondrial fatty acid oxidation (34). Notably, HPSIDF combined with IF treatment reversed HFD-induced changes in lipid composition. Moreover, the content of SMs and CERs was decreased in HFD-fed mice but improved following HPSIDF combined with IF treatment. Several lipidomic studies indicated changes in SMs and CERs in HFD-induced fatty livers. One of the studies showed normal levels of SMs and lower levels of monounsaturated CERs and hexosylceramides in the liver of HFD-fed mice (8). Another study found that CERs and SMs were increased in the fatty livers of rats, and blockade of CER synthesis ameliorated hepatic steatosis (35). Thus, findings on lipid species alterations in the HFD-induced liver were inconsistent. Hence, studies using different experimental setups (i.e., different sample types and preparations, lipidomic identification techniques, etc.) may yield different results (27).

GL metabolism was the most remarkably altered pathway of HPSIDF combined with IF treatment. GLs refer to molecules with glycerol esterified with long-chain fatty acids at any one or all of the hydroxyl groups, which can be divided into monoglycerides, DGs, and TGs according to the number of fatty acids (36, 37). TG is the main form of fat storage in animals, rapidly hydrolyzed to FFAs and DGs by the TG lipases (38). HFD-induced fatty liver is characterized by an increased accumulation of hepatic TG stored in lipid droplets (39). The relative intensities of six TGs [TG (14:0/18:0/20:0), TG (15:0/18:1/22:1), TG (16:0/18:0/20:0), TG (16:0/24:0/18:1), TG (18:0/18:2/20:0), and TG (18:1/18:2/22:4)] in the HPSIDF + IF group were dramatically reduced compared with the HFD group. This phenomenon may be related to the fact that HPSIDF combined with IF treatment alleviates the coalescence of the lipid droplets induced by HFD feeding (40). DGs are activating ligands for the majority of protein kinase C isoforms, serving as substrates for the synthesis of TG and phospholipids, whose synthesis is carefully regulated to coordinate functions in signal transduction (41). Increased DG levels are suspected to be a primary contributor to hepatic lipotoxicity (42). Ruby et al. (43) found an increase in hepatic monounsaturated DGs in obese patients, which correlated with HOMA-IR further underscoring the role of DGs in humans. Interestingly, our study found an increase in DG (18:1/20:3) and a substantial decrease in DG (8:0/18:2) in the livers of HFD-fed mice. Liu et al. (27) confirmed that different DGs might exhibit different trends as structure, saturation, and/or carbon chain length affect biological function. As expected, HPSIDF combined with IF treatment significantly reversed HFD-induced DG changes. Therefore, these six TGs and two DGs may be closely related to the anti-obesity effect of HPSIDF combined with IF treatment.

Next, we investigated the effect of HPSIDF combined with IF treatment on GP metabolism in the livers of HFD-fed mice. Although metabolites of GP were significantly changed in HFD-induced mice compared with the ND group, these changes were reversed by HPSIDF combined with IF treatment. When contrasted with the HFD group, we observed that the levels of PC (15:1/19:1) were dramatically enhanced in the HPSIDF + IF group. PC, a polyunsaturated fatty acid compound, plays a crucial role in the assembly and secretion of very low-density lipoproteins (VLDLs) (44). Impaired hepatic PC biosynthesis remarkably reduces circulating VLDLs and HDL levels, leading to lipid accumulation (45). This implicated that HPSIDF combined with IF treatment could alleviate lipid accumulation by increasing PC (15:1/19:1) to promote higher circulating levels of VLDLs and HDL. Notably, the levels of PC (15:1/20:1) were significantly reduced in the HPSIDF + IF group when contrasted with the HFD group. PC molecules have been reported to contain a range of fatty acyl chains with varying lengths and double-bond positions (21). The secretion of VLDLs in hepatocyte membranes is affected by PC levels and the fatty acyl chain composition of PCs (46). Rong et al. (47) found that deletion of LPCAT3 reduced the number of arachidonic acyl-chains and impaired VLDL lipidation and secretion when arachidonic acid was incorporated into the sn-2 position of PC, leading to hepatic TG accumulation. Thus, the effect of PCs with different acyl chain compositions on the secretion of VLDLs requires further study. Moreover, our results indicated that HPSIDF combined with IF treatment significantly increased the levels of several GPs, including PS (16:0/22:6), LPS (20:1/0:0), and PMeOH (22:5/22:6). Although numerous studies have indicated the effects of GP metabolism on obesity, little is known about how the fatty acid composition of many lipid species affects their distribution and the susceptibility of organisms to obesity (48). Therefore, the specific functions of different GPs may need to be further explored.

Based on the set criteria (FC ≥ 2 or ≤0.5, VIP ≥ 1), 15 differentially regulated lipid species (six TGs, two DGs, one CAR, two PCs, one PS, one LPS, one PMeOH, and one CE) were considered potential lipid biomarkers supported by the HPSIDF combined IF treatment. Spearman correlation analysis showed that TG (18:1/18:2/22:4), TG (15:0/18:1/22:1), TG (14:0/18:0/20:0), TG (18:0/18:2/20:0), TG (16:0/18:0/20:0), and PC (15:1/20:1) were positively correlated with the obese phenotype. The lipid biomarkers screened in this study and their level distribution differed from those reported in previous research. As mentioned earlier, different experimental setups could provide explanations for this phenomenon. Hence, even though specific lipids may appear to act as biomarkers for HFD-induced obesity, these results require further validation in future studies.

We also explored the effect of HPSIDF combined with IF treatment on the expression of genes related to lipid metabolism in the liver of HFD-fed mice. AMPK is an important signaling molecule that regulates hepatic lipid metabolism and contains -α, -β, and -γ subunits. The AMPK-α subunit contains a catalytic phosphorylation site (Thr172) at its NH2 terminus, known to activate AMPK (49, 50). When AMPK is activated, it inhibits lipid accumulation by inhibiting the enzymatic activities of ACC and SREBP-1c, respectively. As a transcription factor, SREBP-1c promotes FFA synthesis by mediating the expression of ACC, SCD1, and FAS downstream genes (51). Our findings demonstrated that HPSIDF combined with IF treatment suppressed the expression levels of SREBP-1c, ACC, FAS, and SCD1 to inhibit TG accumulation and FFA biosynthesis. This is consistent with the reduction in the total content of TGs, DGs, and FFAs in hepatic lipidomic analysis. It is known that activation of AMPK may increase FFA oxidation by stimulating PPARα- and CPT1a-mediated gene expression (52). We found that HPSIDF combined with IF treatment could remarkably up-regulate the mRNA expression levels of AMPKα, PPARα, and CPT1a, thereby accelerating FFA oxidation. Moreover, hepatic cholesterol metabolism is closely related to TG metabolism. Cholesterol biosynthesis is controlled by a cascade pathway in which HMGCR acts as rate-limiting (53). LDLR is another protein involved in cholesterol biosynthesis, and its primary function is to increase LDL reabsorption and reduce hepatic cholesterol synthesis. CYP7A1 is a key enzyme in converting cholesterol to BA and is regulated by LXR (54). In the present study, HPSIDF combined with IF treatment not only significantly inhibited the expression levels of HMGCR, but also enhanced the expression levels of LDLR, CYP7A1, and LXR, which reduced cholesterol biosynthesis and stimulated the conversion of cholesterol to BAs, thereby reducing the serum and liver TC levels. This result is consistent with the elevated BAs content observed in the hepatic lipidomic analysis. Hence, these results suggested that the improvement of metabolic parameters in HFD-fed mice by HPSIDF combined with IF treatment might be due to the modulation of lipid synthesis and metabolism, affecting the hepatic lipidomic profiles.

The present study has several limitations that should be acknowledged. Firstly, the sample size in the lipidomics analysis was small and there was a certain degree of individual variability. The sample size should be sufficiently expanded in future studies to ensure the reliability of the results. Secondly, there is a lack of enrichment (e.g., nesting materials or toys) or monitoring of stress in animals to ensure the welfare of experimental animals and to avoid interference from other factors. All experimental designs should be further developed to ensure the reproducibility of the experiments. Thirdly, this study currently explores the synergistic anti-obesity effect of HPSIDF combined with IF treatment only at the experimental animal level. In future studies, we expect that the superior anti-obesity effect of HPSIDF combined with IF treatment can be demonstrated through clinical trials. Despite these limitations, the results of this study are significant and provide a solid foundation for future research.

## 5 Conclusion

Our study demonstrates that HPSIDF combined with IF treatment can alleviate metabolic abnormalities in HFD-induced mice. These beneficial effects can be attributed to the modulation of levels of multiple liquids, such as TGs, DGs, PCs, PS, and CE in the liver, as shown by lipidomic analysis. Lipidomics profiling and gene expression analysis offer new insights into the anti-obesity effect of HPSIDF combined with IF treatment. Overall, this study provides a theoretical basis for the development of mature fiber functional products, at the same time provides a referable dietary strategy that plays a positive role in improving the comprehensive utilization of okara.

## Data Availability

The raw data supporting the conclusions of this article will be made available by the authors without undue reservation.

## Footnotes

^1^ https://sciex.com.cn/

^2^ http://www.Metaboanalyst.ca

## Glossary

IDF: insoluble dietary fiber
HPSIDF: high-purity insoluble dietary fiber from okara
IF: intermittent fasting
HFD: high-fat diet
TC: total cholesterol
TG: triacylglycerol
LDL-C: low-density lipoprotein cholesterol
HDL-C: high-density lipoprotein cholesterol
FFA: free fatty acid
ELISA: enzyme-linked immunosorbent assay
MeOH: methanol
MTBE: tert-butyl methyl ether
qRT-PCR: quantitative real-time polymerase chain reaction
NAS: NAFLD activity score
PCA: principal components analysis
OPLS-DA: orthogonal partial least squares-discriminant analysis
SREBP-1c: sterol regulatory element-binding protein-1c
ACC: acetyl-CoA carboxylase
FAS: fatty acid synthase
SCD1: stearoyl-CoA desaturase-1
AMPKα: AMP-activated protein kinase-α
RRARα: peroxisome proliferators-activated receptor-α
CPT1a: carnitine palmitoyltransferase-1a
HMGCR: HMG-CoA reductase
LDLR: low-density lipoprotein receptor
CYP7A1: cholesterol 7α-hydroxylase
LXR: liver X receptor
VLDLs: very low-density lipoproteins
