# Supplementary material for: Hepatic lipidomics analysis reveals the anti-obesity effects of insoluble dietary fiber from okara combined with intermittent fasting treatment in high-fat diet-fed mice
Source: Front Nutr. 2025 Apr 23;12:1549105. doi: 10.3389/fnut.2025.1549105 (PMC12055546; doi:10.3389/fnut.2025.1549105)
Supplement: Supplementary file 1 [file Table_1.docx]

**Hepatic Lipidomics Analysis Reveals the Anti-obesity Effects of Insoluble Dietary Fiber from Okara Combined with Intermittent Fasting Treatment in High-Fat Diet-Fed Mice**

Sainan Wang^a,b^, Jiarui Zhang^a,b^, Jiaxin Li^b,c^, Junyao Wang^a,b^, Bo Lyu^a,b^, Wendan Jing^a,b^, Hansong Yu^a,b *^,

Zhao Zhang^d^

^a^ College of Food Science and Engineering, Jilin Agricultural University, Changchun, 130118, China

^b^ Division of Soybean Processing, Soybean Research & Development Center, Chinese Agricultural Research System, Changchun, 130118, China

^c^ Universidade de Vigo, Nutrition and Bromatology Group, Department of Analytical and Food Chemistry, Faculty of Sciences, Ourense, 32004, Spain

^d^ Shandong Sinoglory Health Food Co., Ltd., Liaocheng, 252000, China.

**Supplementary methods**

**Preparation of IDF**

Crude dietary fiber (CDF) with 60% insoluble dietary fiber (IDF) content was provided by Shandong Sinoglory Health Food Co., Ltd. (Liaocheng, China), which was extracted from okara while the hull, oil, and proteins were removed. The CDF was used to prepare the high-purity IDF (90.5%) by the method of Zhao et al. (2017) with some modifications. Briefly, 5 g of CDF was diluted with de-ionized water (1:50 w/v) and subjected to sequential enzymatic digestions, first with 0.5 mL of heat-stable α-amylase (500 U g^-1^, 95-100 °C, 100 rpm water bath for 35 min) and then 1.5 mL of neutral protease (300 U g^-1^, 60 °C, 100 rpm water bath for 30 min). Then, 50 mL of glacial acetic acid and 2 mL of amyloglucosidase (200 U g^-1^, pH = 4.5, 60 °C, 100 rpm water bath for 30 min) were added to the solution. When enzymolysis was over, distilled water (1:4 v/w) at 70 °C was added and stood for 1-1.5 h. The mixture was centrifuged (3500 rpm, 30 min), precipitated overnight with 4 times the volume of 95% ethanol, and vacuum filtered. Finally, the residue was then freeze-dried.

The structure of high-purity IDF was assessed using scanning electron microscopy (SEM; Shimadzu, Tokyo, Japan), X-ray diffraction (XRD; ASX, Brock, Germany), Fourier transform infrared spectroscopy (FT-IR; Shimadzu, Kyoto, Japan) and HPLC (Shimadzu, Kyoto, Japan). IDF had a loose and porous structure, polysaccharide functional groups, and a typical crystalline cellulose I structure. The main monosaccharides of IDF identified were galactose, arabinose, xylose, rhamnose, and glucose. The details on chemical information of IDF were shown in Table. S1 and Fig. S1.

**References**

Zhao, G., Zhang, R., Dong, L., Fei, H., & Zhang, M. (2017). Particle size of insoluble dietary fiber from rice bran affects its phenolic profile, bioaccessibility and functional properties. *LWT-Food Science and Technology*, 87, 450-456.

**Supplementary tables**

**Table S1. The basic composition of IDF**

| Sample | Protein | Starch | Moisture | Ash |
| --- | --- | --- | --- | --- |
| CDF | 15.40 ± 1.11 a | 3.98 ± 0.78 a | 8.30 ± 1.67 a | 1.22 ± 1.88 a |
| IDF | 3.12 ± 0.97 b | 0 b | 3.50 ± 1.83 b | 1.12 ± 1.76 a |

CDF, crude soybean dietary fiber; IDF, high-purity insoluble dietary fiber from okara. Values are expressed as g per 100 g dry matter (mean values). Different letters in the same column (a and b) are significantly different (*p* < 0.05). The results are expressed as mean ± SD (n = 3).

**Table S2. Composition and energy distribution of experimental diets**

| Ingredient | D12450B *^a^* | | D12492 *^b^* | |
| --- | --- | --- | --- | --- |
|  | gm | kcal | gm | kcal |
| Casein, 80 Mesh | 200 | 800 | 200 | 800 |
| L-cystine | 3 | 12 | 3 | 12 |
| Corn starch | 315 | 1260 | 0 | 0 |
| Maltodextrin 10 | 35 | 140 | 125 | 500 |
| Sucrose | 350 | 1400 | 68.8 | 275.2 |
| Cellulose, BW200 | 50 | 0 | 50 | 0 |
| Soybean oil | 25 | 225 | 25 | 225 |
| Lard | 20 | 180 | 245 | 2205 |
| Mineral Mix S10026 | 10 | 0 | 10 | 0 |
| Dicalcium Phosphate | 13 | 0 | 13 | 0 |
| Calcium Carbonate | 5.5 | 0 | 5.5 | 0 |
| Potassium citrate,1 H20 | 16.5 | 0 | 16.5 | 0 |
| Vitamin Mix V10001 | 10 | 40 | 10 | 40 |
| Choline Bitartrate | 2 | 0 | 2 | 0 |
| FD&C Red Dye #40 | 0.05 | 0 | 0.05 | 0 |
| Total | 1055.05 | 4057 | 773.85 | 4057 |
|  | gm% | kcal% | gm% | kcal% |
| Protein | 19.2 | 20 | 26.2 | 20 |
| Carbohydrate | 67.3 | 70 | 26.3 | 20 |
| Fat | 4.3 | 10 | 34.9 | 60 |
| Total |  | 100 |  | 100 |
| kcal/gm | 3.85 |  | 5.24 |  |

*^a^* D12450B, a normal diet with 4.30% fat content (10% fat calories); *^b^* D12492, a high-fat diet with 34.90% fat content (60% fat calories).

**Table S****3. Sequences of primers used in qRT-PCR**

| Genes | Forward primer (5’-3’) | Reverse primer (5’-3’) |
| --- | --- | --- |
| β-actin | GGCTGTATTCCCCTCCATCG | CCAGTTGGTAACAATGCCATGT |
| AMPKα | GGAGGACAATGACGAAGACGGATG | TCTGGAGGAAGAGATGGAGTGATGG |
| SREBP-1c | GCTACCGGTCTTCTATCAATGA | CGCAAGACAGCAGATTTATTCA |
| ACC | AGGAGGAGGATGGCAGAAGTTGAG | AAGCAGAGTCGCAGCATGAATGG |
| FAS | CAAGTGCAAACCAGACTTCTAC | GCACTTTCTTTTCCGGTACTTT |
| SCD1 | AACATTCAATCCCGGGAGAATA | GAAACTTTCTTCCGGTCGTAAG |
| PPARα | CCAGATGTGCCTGCTGCTTCC | TGGTCGGTCTACAGAGTGAGTTCC |
| CPT1a | AGGAGGAGGATGGCAGAAGTTGAG | AAGCAGAGTCGCAGCATGAATGG |
| HMGCR | ATACAAGTATAGCTGGACGCAA | CTGCATTTCAGGGAAATACTCG |
| LDLR | CTGTAGGGGTCTTTACGTGTTC | GTTTTCCTCGTCAGATTTGTCC |
| CYP7A1 | GGAAAACCTCCAACGTATCATG | GGAAAGACTTTGTCGAATTGCT |
| LXR | GAGTGTCGACTTCGCAAATG | CTTCAGTTTCTTCAAGCGGATC |

**Supplementary figures**


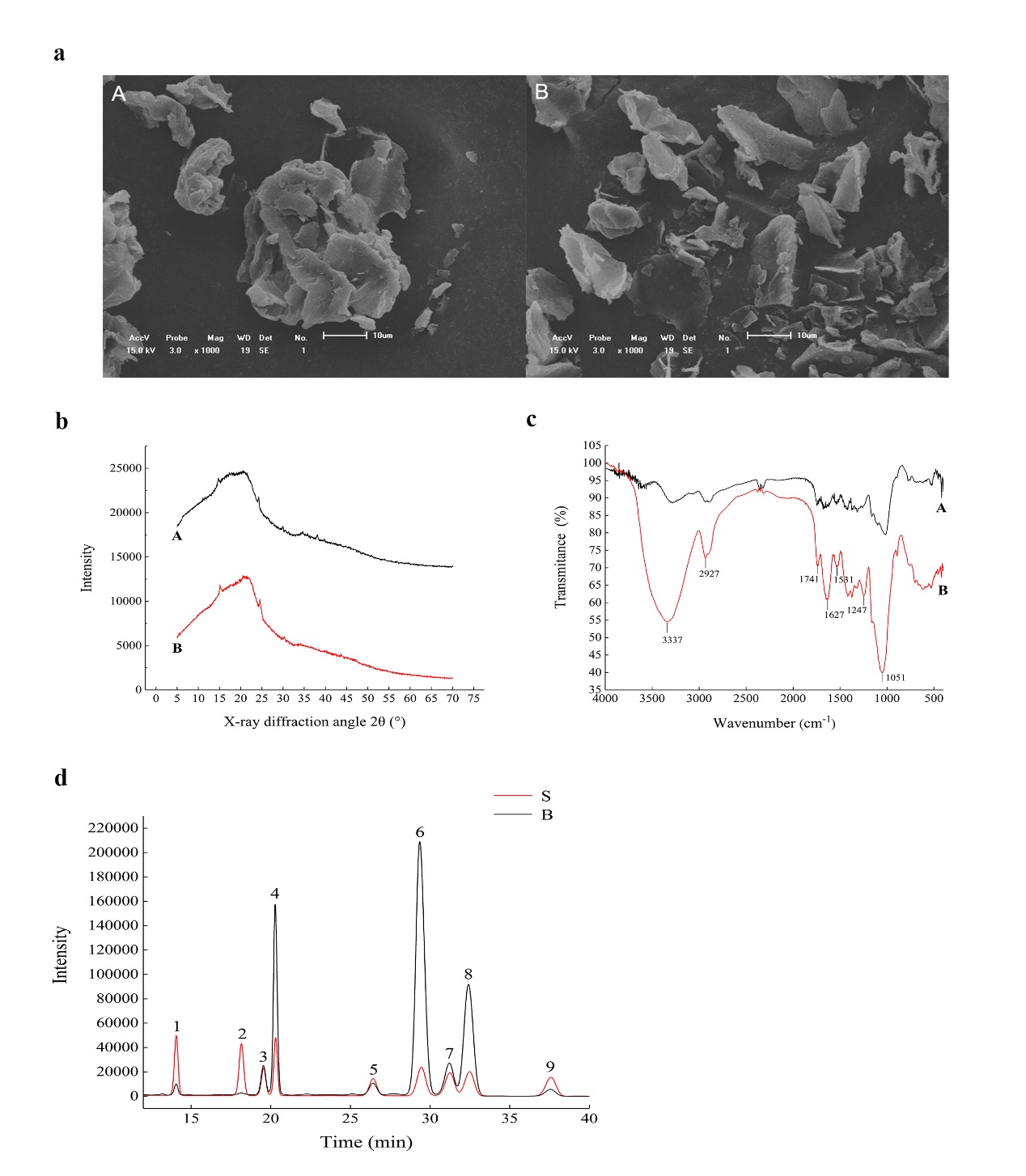


**Figure S1. The structure and composition of IDF.** (a) Scanning electron microscopy (SEM) analyses of CDF (A) and IDF (B) at 1000× magnification. (b) X-ray diffraction (XRD) spectra of CDF (A) and IDF (B). (c) Fourier transform infrared spectroscopy (FT-IR) of CDF (A) and IDF (B). (d) The monosaccharide composition of IDF. S, standard; B, IDF; Numbers 1 to 9 represent the standards of mannose, glucuronic acid, rhamnose, galacturonic acid, glucose, galactose, xylose, arabinose, and fucose respectively.
